# Liver Transplantation Using a Graft from a Donor with Situs Inversus Totalis: A Case Report and Review of the Literature

**DOI:** 10.1155/2013/532865

**Published:** 2013-09-10

**Authors:** Xu-Yong Sun, Ke Qin, Jian-Hui Dong, Hai-Bin Li, Liu-Gen Lan, Ying Huang, Song Cao, Zhuang-Jiang Li

**Affiliations:** Guangxi Key Laboratory of Transplant Medicine, Institute of Transplant Medicine, 303 Hospital of People's Liberation Army, Nanning 530021, China

## Abstract

It is critical to effectively use every available organ to meet the increasing demands for liver transplantation. Situs inversus is a rare congenital anomaly caused by obstruction of viscus rotation during embryonic development. Situs inversus was once regarded as a contraindication to liver transplantation because of the technical difficulties associated with the unique vascular anatomy and concern about achieving accurate graft positioning. Here, we present a successful case of liver transplantation using a graft from a donor with situs inversus totalis. The related experience will contribute to opening up new realms for the use of such rare organ resources.

## 1. Introduction

Situs inversus is a rare congenital anomaly caused by obstruction of viscus rotation during embryonic development. Its incidence is about 0.01% worldwide. The viscus distribution in the situs inversus individuals is opposite to ordinary people. All thoracoabdominal organs and retroperitoneal organs (i.e., kidneys and adrenal glands) are symmetrically positioned to the midline, which is often called mirror-image reversal [1]. Situs inversus was once regarded as a contraindication to liver transplantation because of the technical difficulties associated with the unique vascular anatomy and concern about achieving accurate graft positioning [2, 3]. For a number of patients with situs inversus, liver transplantation has been successfully performed during the last two decades [4–8]. However, few cases of deceased donor organs with situs inversus used for liver transplantation have been reported [3, 9–11]. Here, we present a case of liver transplantation using a graft from a donor with situs inversus totalis.

## 2. Case Report

A 23-year-old male was referred to our hospital in May 2008 due to severe traumatic brain injury caused by a traffic accident and was diagnosed as brain dead shortly after admission. Dextrocardia was found during physical examination and situs inversus totalis was verified by further X-ray and ultrasonic inspection. During the retrieval of donor organs, it was found that the liver, pancreas, and stomach were located in the opposite positions. The descending aorta was located directly in front of the spine and the inferior vena cava was found in the front left of the spine. Organ procurement was smoothly performed without great difficulty even though the procedure took a little longer due to the operator's habitual thinking about the anatomy. During preparation of the donated liver, the anatomical relations were clearly identified, that is, liver artery locating in right side and common bile duct in the left, and portal vein still being behind common bile duct and liver artery. The hepatic superior and inferior vena cava was cut to angle well in preparation for vascular anastomosis.

A 58-year-old female recipient was diagnosed with end-stage liver disease caused by type B hepatitis. Preoperative condition was evaluated with Child-Pugh (class A) and MELD (4.3). The liver transplantation was performed in May 2008. The incision was made double subcostally. Following isolation of porta hepatis and hepatic ligament, the retrohepatic inferior vena cava was partially clamped and then the diseased liver was removed preserving the inferior vena cava. The right hepatic vein was suture ligated while preserving the left and middle hepatic veins. At the point of left hepatic vein entering the hepatic superior and inferior vena cava, a transection was taken on an anterior wall of the interval between the left and middle hepatic veins, and meanwhile, the anterior wall of inferior vena cava was cut downward 2 cm, thereby forming a “T” type anastomotic rim in preparation for hepatic venous outflow reconstruction.

**Figure 1:**
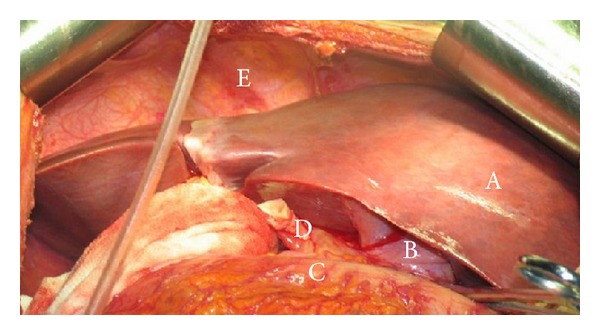
The status of transplanted liver. Orthotopic liver transplantation was performed with a graft from a donor with situs inversus totalis. Piggyback technique was used for the transplantation. The photograph was taken prior to the reconstruction of hepatic artery and postreconstruction of portal vein. (A) Liver; (B) gallbladder; (C) colon; (D) hepatic artery; (E) diaphragm.

Liver transplantation was performed with a modified piggyback technique. The donor liver was directly placed into the recipient's right upper quadrant without rotating adjustment, that is, the right hepatic lobe was positioned in the recipient's left side and the smaller lobe was positioned in the recipient's liver fossa. The hepatic superior and inferior vena cava was directly anastomosed to recipient's inferior vena cava with 3-0 vascular suture. Portal veins were end-to-end anastomosed with 6-0 vascular suture followed by albumin solution perfusion. The residual UW solution (University of Wisconsin solution) in the donated liver was flushed away with albumin solution. Then, the intrahepatic vena cava was completely ligated and the clamps were removed from portal vein and vena cava, thereby ending an hepatic phase. Cold and warm ischemia times were 7 hr and 2 min, respectively. For artery reconstruction, the cut off at the junction of the proper hepatic artery and gastroduodenal artery (recipient) was anastomosed with the cut off at the junction of common hepatic artery and splenic artery (donor) with 7-0 vascular suture. Biliary reconstruction was performed by duct-to-duct anastomosis between recipient's common hepatic duct and donor's common bile duct with 6-0 vascular suture without placing a “T” type drainage tube. The total operation time was 6.5 hr and life support was withdrawn at 6 hr after operation.  Figure 1 illustrates the status of the transplanted liver after reconstruction of the portal vein. The immunosuppression was induced with anti CD25 antibody and maintained with FK506/MMF. The patient recovered well postoperatively with normal hepatic function in 2 weeks and discharged from hospital at day 20 after transplantation. By now, the patient has been followed up for 36 months. The liver function remains normal without biliary complication and rejection reactions. 

## 3. Discussion

The shortage of donated organs has been a critical issue with regard to the increasing demand. In order to enhance usable organ sources, people try to expand the criteria for donor evaluation and attempt the maximum possible use of every donor resources. Situs inversus totalis is a very rare anatomical abnormality, which is characterized by dextrocardia and “mirror-image” distribution of abdominal organs. It may coexist with other malformations such as underdevelopment of portal vein and inferior vena cava, polysplenia syndrome, and congenital biliary atresia [12]. Situs inversus totalis was considered as an absolute contraindication in organ transplantation mainly due to the anatomic deformities of viscera and blood vessels. Along with the extensive implementation and technological advance of liver transplantation, a number of ELD patients with situs inversus totalis had successful liver transplants, and few patients received liver implantation from donors with situs inversus totalis. However, a standard operation procedure has not been established in terms of this type of liver transplantation.

In 1995, Asfar et al. [9] reported a case of liver transplantation using a graft from a donor with situs inversus totalis. A 23-year-old female DBD (donation after brain death) donor was identified with situs inversus totalis and dextrocardia. The recipient was a 63-year-old male who suffered from alcoholic cirrhosis. During the operation, the liver was counter-clockwise rotated at 90°, that is, left side of the liver was located in recipient's right subphrenic space and the right side of the liver was placed in paracolic sulci. The hepatic inferior vena cava was ligated followed by an end-to-side anastomose between the intrahepatic vena cava and recipient's inferior vena cava and end-to-end anastomoses between portal veins and hepatic arteries. Youx-en-Y biliary anastomosis was performed for biliary reconstruction. Liver function was restored in 1 week following liver implantation. The patient died from ARDS and septicemia at day 20 after the operation. This case indicated the feasibility of liver transplantation using a graft from a donor with situs inversus totalis even though long-term survival was not achieved on this recipient. In another two cases using liver grafts from donors with situs inversus totalis, orthotopic liver transplantation was performed with piggyback technique [10, 13]. The liver was implanted in the original orientation without adjustment and recipients achieved long-term survival. In Pomposelli et al.'s report, piggyback technique was also used for orthotopic liver transplantation, but the donor liver was backward rotated at 180° along the axis of inferior vena cava allowing a big part of the left side of the liver to be located in the right upper abdominal cavity. After the directional adjustment, the common bile duct of the graft was toward the up front, and Roux-en-Y biliary intestinal anastomosis was carried out for biliary reconstruction. The long-term survival of the patient was achieved following liver transplantation. In 2011, a modified piggyback technique was used in orthotopic liver transplantation with a situs inversus liver [14]. Recipient's right hepatic vein was suture ligated and a “T” type anastomotic rim composed of left and middle hepatic veins and inferior vena cava was created for hepatic venous outflow of the transplanted liver. Recently, Dou et al. [15] successfully performed a classical orthotopic liver transplantation from a donor with situs inversus totalis. The liver graft was placed in the recipient's right upper abdominal cavity with a slightly rightward rotation. After finishing the conjunctions of suprahepatic and intrahepatic inferior vena cava, the interspace was filled with omentum majus. The implanted liver was fixed to the diaphragm with falciform ligament.

In the present report, the donor with situs inversus totalis died from severe traumatic brain injury caused by-traffic accident. The distribution of viscera was confirmed with X-ray and type-B ultrasonic checks, and a planned procedure was laid out before the organ retrieval. In consideration of the recipient being at the end-stage of liver disease without cancerization, a modified piggyback technique was used for the orthotopic liver transplantation. During operation, the liver graft was slightly rightward rotated and a water bag was temporarily placed in the liver fossa supporting the graft in order to avoid its self rotating. After the removal of the diseased liver, there was enough space in right upper quadrant to hold the liver graft, and vascular bypass was not required. The inferior vena cava involved a “T” type anastomotic rim which ensured the hepatic venous outflow to be unobstructed. The hepatic artery was closely related to bile duct, which was adjusted by slightly changing liver position during anastomosis of vascular and biliary systems. End-to-end anastomosis was applied for the reconstruction of hepatic artery and biliary duct. Since the left hepatic lobe was relatively large, the liver graft was laid up in situ placement. The remaining space in the right upper quadrant was filled with omentum majus and the liver was mounted to the diaphragm with falciform ligament. This manipulation could protect the liver from graft self-rotation without repressing the stomach and the surrounding intestinal tract. The achievement of long-term survival proved the rationality of the planned procedure in this particular case.

In the face of critical shortage of donor livers, we treasured every valuable donor resource to save as many lives as possible. All donors received routine auxiliary examinations and the condition of the anticipated organ was predetermined prior to organ procurement. Especially to liver graft with situs inversus, timely awareness of the exceptional situation and a detailed procedure in advance are prerequisites to the success of transplantation. During donor liver retrieval, the location and relationship among various vessels need to be carefully distinguished to prevent operation damage to the organ. During implantation of the liver graft, the following two aspects should be seriously considered, the reconstruction of hepatic venous outflow which is not to be obstructed and fixation of liver graft to prevent self-rotation. Thus to our experience, the success of transplantation using such infrequently donated organs could be achieved through careful preoperative examination and an elaborately planned operation procedure.
